# A UDF-Based Approach for the Dynamic Stall Evaluation of Airfoils for Micro-Air Vehicles

**DOI:** 10.3390/biomimetics9060339

**Published:** 2024-06-04

**Authors:** Diana-Andreea Sterpu, Daniel Măriuța, Lucian-Teodor Grigorie

**Affiliations:** 1 Faculty of Aerospace Engineering, POLITEHNICA National University of Science and Technology Bucharest, 060042 Bucharest, Romania; sterpudianaandreea@gmail.com; 2 Department of Aircraft Integrated Systems and Aviation, Military Technical Academy “Ferdinand I” Bucharest, 050141 Bucharest, Romania; daniel.mariuta@mta.ro

**Keywords:** aerodynamics, hysteresis, NACA 0012, DES

## Abstract

A numerical method for generating dynamic stall using ANSYS Fluent and a user-defined function (UDF), with the complete script shared for reference, is introduced and tested. The study draws inspiration from bird flight, exploring dynamic stall as a method for achieving enhanced aerodynamic performance. The numerical method was tested on NACA 0012 airfoils with corresponding chord lengths of $c_{1}=40\;mm$, $c_{2}=150\;mm$, and $c_{3}=300\;mm$ at Reynolds numbers ranging from ${Re}_{1}=2.8\times 10^{4}$ up to ${Re}_{5}=1.04\times 10^{6}$. Airfoil oscillations were settled for all cases at ω=0.55 Hz. Detached eddy simulation (DES) is employed as the turbulence model for the simulations presented, ensuring the accurate representation of the flow characteristics and dynamic stall phenomena. The study provides a detailed methodology, encouraging further exploration by researchers, especially young academics and students.

## 1. Introduction 

The flight of birds has inspired research and experiments in aerodynamics since the first human attempt at air transportation, successfully achieved by the pioneers of the first practical airplane, the Wright brothers [1,2,3,4]. Approaches to aerodynamic imitation of bird flight vary from thrust generation by airfoil oscillations [3] to state-of-the-art shape morphing methods [4,5,6,7,8]. 

One method that is explored to fulfill the human aspiration of soaring like birds involves the examination of dynamic stall and its associated advantages. Dynamic stall, a phenomenon captivating aerodynamic research for over five decades, involves rapidly changing a wing’s angle of attack (AoA) to create a high lift moment. Thus, dynamic stall is the aerodynamic phenomenon used by long-distance flying birds to achieve great aerodynamic performance in a short period of time by rapidly changing the AoA of their wings in order to create high lift moments. This research aims to bridge past efforts with contemporary accessibility, emphasizing the importance of a practical and reproducible approach.

Dynamic stall was investigated in 1977 through experiments conducted by National Aeronautics and Space Administration (NASA) [6,9], which also introduced a software package to advance the research further. This research provided empirical data and insights into the phenomenon and encouraged further study [6,10,11]. While acknowledging the importance of past research, software packages, and methods, it is imperative to embrace a contemporary and accessible approach for replicating dynamic stall. This is crucial to inspire and engage young academics and students, encouraging exploration of the advantages of dynamic stall and potentially uncovering new insights. The progress of dynamic stall research has been closely intertwined with the progress in the computational fluid dynamics (CFDs) commercial tools thatwha are available today (e.g., ANSYS Fluent, OpenFOAM, Autodesk CFD, etc.). Recent advancements in numerical solutions for unsteady inviscid and viscous flows have enabled the detailed visualization of essential flow processes in numerical wind tunnels. This article provides a detailed reproduction method for dynamic stall, aiming to enhance accessibility for researchers to a validated approach and to foster innovation and information sharing within the global research community.

The exploration of dynamic stall not only sheds light on fundamental aerodynamic phenomena but also holds promise for practical applications, particularly in the realm of biomimetics. As proposed in [12,13,14], the integration of pitching and plunging motions of an airfoil for thrust generation represents a compelling avenue for innovation. This concept finds application across various domains, including solar energy generation, where dynamic stall phenomena play a crucial role in optimizing the efficiency of turbine blades. Additionally, in aerospace engineering, the exploration of dynamic stall in pitching and plunging motions opens avenues for enhancing the maneuverability and performance of UAVs and micro-air vehicles (MAVs). Moreover, in wind energy applications, such as vertical axis wind turbines, dynamic stall behaviors are harnessed to improve energy capture and overall system efficiency. These diverse applications underscore the versatility and potential of dynamic stall studies in advancing technological innovation across different fields. 

Various research papers address the dynamic stall investigation using NASA’s OVERFLOW 2.3 CFD code for delayed detached eddy simulations (DDESs) [15] or ANSYS Fluent CFD code for simulating 2D dynamic stall on fluctuating freestream [16] or for analyzing multi-frequency excitation [17]. Additionally, a variety of experimental approaches have been employed to achieve the same objective [18,19,20]. Predicting airfoil stalling dynamics using numerical solutions [21], combining pitch-plunge oscillations of an airfoil for power production [14], dynamic stall control by airfoil deformation [22], and studying the noise produced on an oscillating airfoil [23] all contribute to the potential applications of dynamic stall in both research and industrial contexts. The main focus regarding recent dynamic stall investigations is placed on rotating wings with helicopter applications [24,25] and vertical axis wind turbine applications [26,27]. 

The main objective of this work is testing a user-defined function in Ansys Fluent to replicate dynamic stall on airfoils suitable for MAVs at relatively high Reynolds numbers. The novelty lies in a more technical approach for elucidating the process of reproducing dynamic stall in ANSYS Fluent with the intention of fostering further studies in this field. In adopting a more practical approach, the intention is to share a tested numerical method, allowing for immediate utilization and potential improvement by researchers for various applications, thereby enhancing the accessibility and applicability of dynamic stall studies in the field of aerodynamics.

## 2. Methodology

The research design involves dynamic stall testing on a range of airfoil geometries utilizing the provided UDF script. By selecting the NACA 0012 and sample-sized airfoils, an easy comparison with existing experimental and numerical results within the research community is facilitated. The objective is to showcase the alignment of the UDF method presented in this paper with findings from prior experimental and numerical investigations. The dissemination of this novel method aims to enhance accessibility to dynamic stall benefits among academics and foster exploration of various suggested applications outlined later in the paper.

### 2.1. Geometry and Mesh Generation

**Figure 1:**
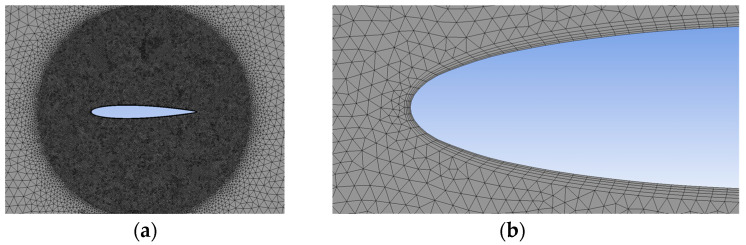
Mesh of NACA 0012 airfoil: (**a**) internal zone; (**b**) inflation layers.

For the current study, NACA 0012 airfoils were selected, with chord lengths of $c_{1}=40\;mm$, $c_{2}=150\;mm$, and $c_{3}=300\;mm$ respectively. The initial geometry places each airfoil at 0° AoA to initiate dynamic stall. The center of gravity (CG), positioned at ½ chord on the mean camber line of each airfoil, represents an essential aspect of the UDF, particularly regarding the initiation of motion. Unstructured meshes, employing triangular elements, were generated to encompass the respective airfoil geometries (see Figure 1). Internal zones were specifically established near each airfoil to capture a more intricate perspective of the pressure distribution. 

Meshing is itself a preliminary step in aerodynamics as well as an interpretation of the studied effect. Critical aspects of this stage involve defining the study’s dimensions, considering the impact of walls on the flow, ensuring that the mesh quality is reflected in the obtained results, and determining the dimensions of the internal zone. Inflation layers serve as a valuable tool for controlling the wall spacing and ensuring appropriate y+ values, where y+ represents the nondimensional distance from the wall to the first grid point. In the simulations conducted for all the NACA 0012 airfoil geometries, including chord lengths of $c_{1}=40\;mm$, $c_{2}=150\;mm$, and $c_{3}=300\;mm$, employing five inflation layers generated from the airfoil yielded satisfactory results, effectively controlling y+ values and ensuring accurate boundary layer resolution.

Considering the turbulence model desired for replicating dynamic stall in this paper (DES), it is necessary to impose careful mesh refinement to adequately resolve both the near-wall regions and the larger turbulent structures. Inadequate mesh resolution can result in numerical inaccuracies, particularly in regions where the flow transitions between the laminar and turbulent states. This sensitivity to mesh resolution can make DESs computationally expensive, especially for simulations involving complex geometries or high Reynolds number flows.

### 2.2. User-Defined Function

A UDF is a C written script compatible with ANSYS Fluent. In the presented case study, the UDF should swiftly rotate the airfoil to induce dynamic stalling benefits, followed by a recalibration to the initial AoA.

In this specific context, the airfoil is regarded as a rigid body, while the mesh is the deformable structure. Hence, in each iteration, the mesh needs to adapt to the updated position of the airfoil. The tracking of iterations is time-dependent, rendering this study unsteady.

#### 2.2.1. Mathematical Approach

The UDF in this study serves as the digital mechanism for inducing dynamic stall benefits. This computational tool lays the groundwork for delving into the mathematical underpinnings governing airfoil dynamics. Within this mathematical framework, the variation of the airfoil’s AoA between a minimum value and a maximum value is defined, establishing a foundation for analysis. This systematic approach enables the elucidation of the intricate dynamics of dynamic stall phenomena with engineering rigor and clarity. The variation of the airfoil AoA is defined between a minimum value ($\alpha_{min}$) and a maximum value ($\alpha_{max}$):

(1) $$∆\alpha =\alpha_{max}-\alpha_{min}.$$

**Figure 2:**
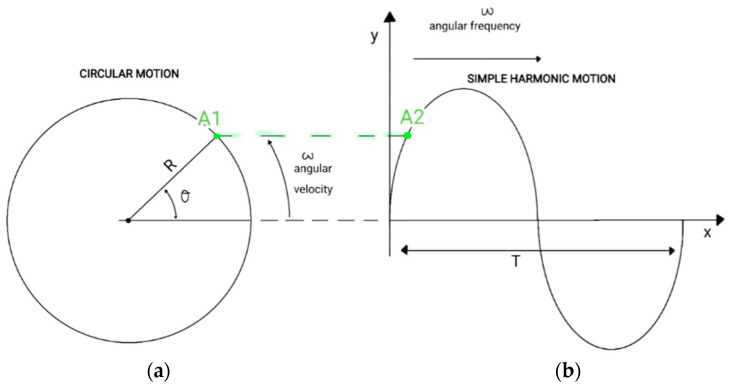
Oscillating motion: (**a**) circular motion; (**b**) simple harmonic motion.

The governing pitching of the airfoil equation must be defined as a time-dependent sinusoidal equation that guarantees the AoA oscillation. For enhanced comprehension, the following scheme represents the transition between the angular velocity ω, defining circular motion, and the angular frequency ω=0.55 Hz, characterizing the simple harmonic motion (SHM) (see Figure 2). 

Following the above scheme, point A1, which defines an element in the circular motion, represents the amplitude determination of point A2, which belongs to the SHM. The following statements can be made in comparing the above representations:

- In circular motion, ω represents the change of angular displacement; in SHM, ω represents the rate of change of the phase angle.
- The circulation motion’s radius R is the SHM amplitude:

(2) y=Rsin⁡ωt.

- The angular velocity, ω, of the circular motion is also the angular frequency, ω, of the SHM.
- The angular frequency (ω) represents how many cycles per second are completed:

(3) $$\omega =\frac{2\pi }{T},$$

where T represents the period (oscillation time).

For this paper, the oscillation frequency is ω=0.55 Hz, a value which is grounded in practical applications and aligns with biomimetic principles observed in natural flight. In biomimetics, where engineering solutions are inspired by biological systems, such frequencies mimic the natural resonant frequencies observed in birds and insects, ensuring a closer emulation of their flight dynamics. Through the integration of biomimetics and aerodynamics, the frequency selection in the present study exemplifies the synergistic relationship between biological inspiration and engineering innovation, paving the way for the development of more efficient and agile aerial systems. 

#### 2.2.2. Programmable Implementation

In its current state, the provided script (see Table 1) produces half of a dynamic stall, covering the motion from 0° to 18° AoA. This selection is based on extensive aerodynamic analyses, considering the critical transitional range where dynamic stall phenomena manifest prominently. The chosen angles of attack are strategically aligned with aerodynamic principles governing lift and flow separation, ensuring the script’s relevance to dynamic stall studies across various airfoil geometries. The linear (vel) and angular (omega) velocities are returned to ANSYS Fluent by overwriting the arrays. The desired result consists in an oscillating airfoil which is pitching from 0° to 18° AoA and back [28,29]. The provided UDF code offers several advantages over conventional CFD tools due to its programmability and adaptability, particularly in handling the complexity of iterations. Unlike standard CFD software, which may have limited capabilities for customizing dynamic stall simulations, this UDF allows for fine control of the iterative process. By directly interfacing with ANSYS Fluent, the code empowers researchers to manipulate thread variables, define custom conditions, and implement specialized algorithms to accurately model dynamic stall phenomena. This level of control enables the tailoring of simulations to specific requirements, optimizing computational resources and reducing unnecessary calculations. Moreover, the code’s logical structure and clarity enhance readability and maintainability, facilitating efficient troubleshooting and code refinement.

Future research endeavors may focus on extending the capabilities of the UDF approach to encompass additional functionalities, thereby broadening its utility in biomimetic-inspired aerodynamic investigations. One potential avenue for enhancement lies in integrating features that account for airfoil deformation, thereby enabling more comprehensive modeling of dynamic stall phenomena with consideration for structural flexibility. Additionally, efforts could be directed towards extending the UDF to support dynamic stall simulations in three-dimensional geometries, facilitating the exploration of complex flow dynamics and interactions in scenarios more closely resembling real-world conditions encountered in both biological and industrial contexts. Furthermore, the UDF could be tailored to simulate aerodynamic interactions in multi-body systems, facilitating investigations into collective behavior and cooperative flight strategies inspired by social insects or flocking birds.

The simulations presented in this paper required moderate computational resources, typically available in standard desktop or workstation environments, facilitating accessibility and reproducibility.

## 3. Results and Discussion

### 3.1. Numerical Results for NACA 0012—$c_{3}=300\;\mathrm{mm}$

**Figure 3:**
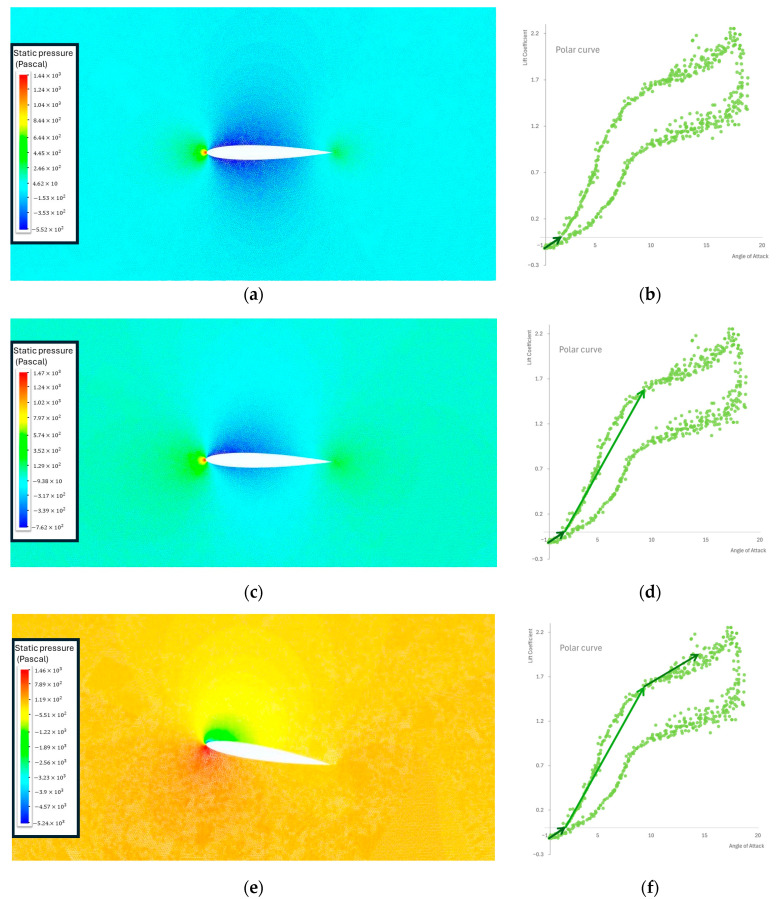

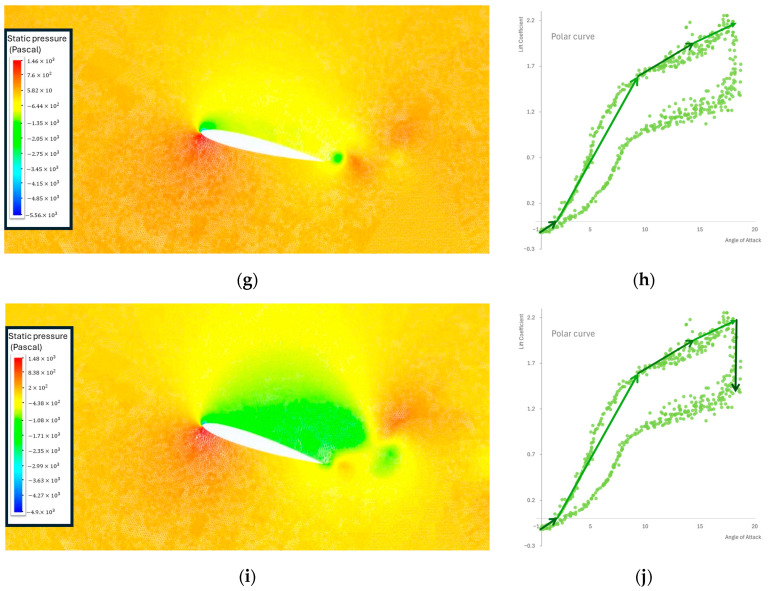
(**a**) Pressure contour at 0° AoA. (**b**) Evolution of the polar curve at 0° AoA. (**c**) Pressure contour at 3.2° AoA. (**d**) Evolution of the polar curve at 3.2° AoA. (**e**) Pressure contour at 9.81° AoA. (**f**) Evolution of the polar curve when vortex forms near the leading edge at 9.81° AoA. (**g**) Pressure contour at 13.08° AoA. (**h**) Evolution of the polar curve when moment stall occurs at 13.08° AoA. (**i**) Pressure contour at 17.98° AoA (**j**) Evolution of the polar curve with significant drag increase caused by the separation of the flow.

Firstly, the UDF was tested on a NACA 0012 profile of larger chord length ($c_{3}=300\;mm$) at ${Re}_{5}=1.04\times 10^{6}$. The results are presented in Figure 3 and serve as an example of the effect of dynamic stall on the pressure contour of the airfoil, juxtaposed with the theoretical polar curve depicting the lift coefficient against the AoA. The pressure contour on the lower contour of the airfoil is slightly bigger than the one on the upper contour, resulting in lift generation (Figure 3c,d). The dynamic-stall vortex is forming at the leading edge of the airfoil. The swift rotation of the airfoil to a AoA does not immediately induce a pressure change on the upper surface corresponding to the new AoA. This delay is attributed to the time lag between the airfoil’s motion and the subsequent pressure response (Figure 3e,f). The dynamic-stall vortex advanced on the upper contour of the airfoil from the leading edge to the trailing edge. The airfoil momentarily reacts to a lower AoA than the geometric AoA at its current state. The delayed response to the geometrical AoA also results in a postponement of the boundary layer separation. The vortex is initiated near the leading edge of the airfoil and progresses toward the trailing edge along the upper contour of the airfoil. During this stage, a substantial additional lift is generated. Once the dynamic stall vortex surpasses the trailing edge, there is a notable reduction in lift (Figure 3g,h). Upon reaching the ultimate stage, leading to complete flow separation (deep stall state), the flow reattachment phase persists as the AoA decreases. The process concludes when the flow is fully reattached to the contour of the airfoil (Figure 3i,j).

The specific behavior of dynamic stall is highly contingent upon the geometry of the airfoil and the Reynolds number to which the airfoil is subjected. With that in mind, the presented results should function as both a guide and a validation of the methodology outlined in this study.

### 3.2. Numerical Results for NACA 0012—$c_{2}=150\;\mathrm{mm}$

**Figure 4:**
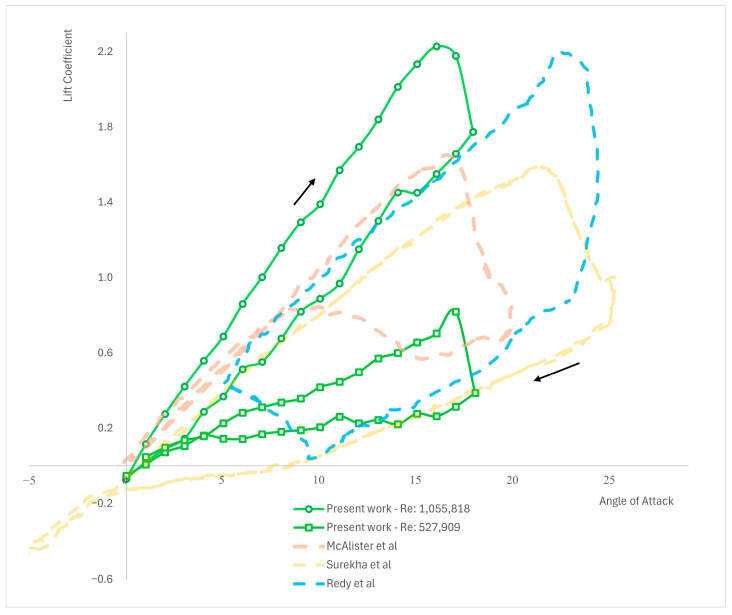
Comparison of the results for NACA 0012, *c*_2_ = 150 mm of this paper against experimental results from Reddy et al. (1987) [11]; Surekha et al. (2019) [18]; and McAlister et al. (1978) [6].

In this section, the results obtained from the simulations using the NACA 0012 airfoil with a $c_{2}=150\;mm$ chord length at ${Re}_{4}=5.2\times 10^{5}$ and ${Re}_{5}=1.04\times 10^{6}$ are presented (see Figure 3 and Figure 4). The investigation reveals that the results obtained through the utilization of the UDF exhibit behavior consistent with the experimental findings (as compared in Table 2 and graphically presented in Figure 4). Notably, the uplift curve demonstrates a higher lift coefficient compared to any of the other experimental results. However, such disparities are commonly encountered and deemed normal in aerodynamic analyses. It is essential to note that the experimental results were conducted on diverse geometries and AoAs, which are comprehensively presented in Table 2.

**Figure 5:**
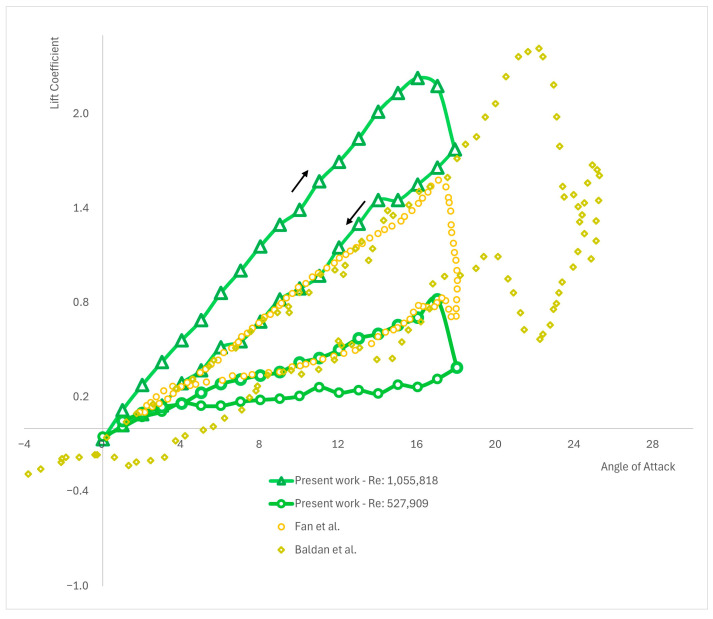
Comparison between current work results for NACA 0012, $c_{2}=150\;mm$ and CFD results obtained by Fan et al. (2019) [30], and Baldan et al. (2023) [31].

The following Table 3 and Figure 5 illustrate the testing of the UDF-obtained results against the numerical results from other studies. Comparing numerical results is crucial to validate the accuracy of the presented UDF, which is functioning within ANSYS Fluent, ensuring that the findings are reliable and consistent with established data.

### 3.3. Numerical Results for NACA 0012—$c_{1}=40\;\mathrm{mm}$

**Figure 6:**
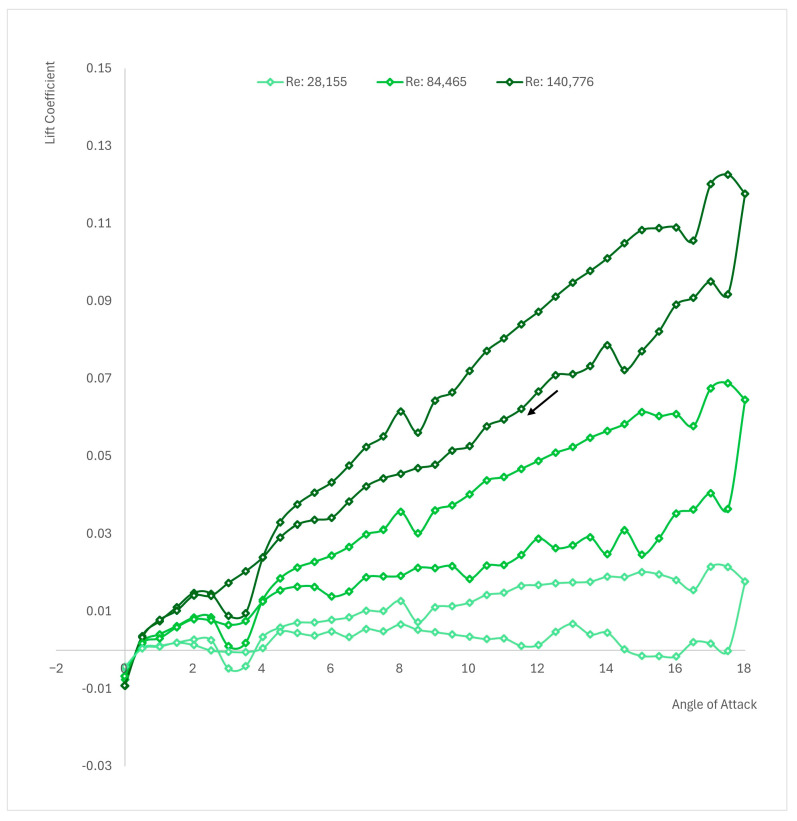
Polar curve of NACA 0012 for $c_{1}=40$ mm at ${Re}_{1}=2.8\times 10^{4}$, ${Re}_{2}=8.4\times 10^{4}$, and ${Re}_{3}=1.4\times 10^{5}$.

The examination of the $c_{1}=40\;mm$ chord length NACA 0012 airfoil, as well as micro airfoils in general, is a crucial aspect of aerodynamic research, particularly concerning MAVs, drones, and other small-scale aerial platforms. These airfoils play a vital role in determining key factors like maneuverability, stability, and efficiency. Figure 6 illustrates the outcomes obtained from the examination of the NACA 0012 airfoil with a chord length of $c_{1}=40$ mm undergoing dynamic stall under various Reynolds numbers, ${Re}_{1}=2.8\times 10^{4}$, ${Re}_{2}=8.4\times 10^{4}$, and ${Re}_{3}=1.4\times 10^{5}$. Additionally, drawing inspiration from the efficient aerodynamics observed in small flying creatures such as insects or birds contributes valuable insights to the design and improvement of micro airfoils used in MAVs or drones.

Analyzing Figure 6, it can be observed that the lower the Reynolds number, the lower the lift slope is. At ${Re}_{1}$, it can be observed that the lift coefficients are close to zero for the return line. The highest lift slope is obtained at ${Re}_{3}$. Overall, if the lift coefficients from all the three cases are analyzed, it can be observed that lift coefficients decrease when the chord length is decreased. By incorporating DES into the numerical simulations of micro airfoils, this study enhances the fidelity and accuracy of the results obtained. Specifically, DES enables the capture of both near-wall turbulence and large-scale unsteady flow structures, providing a comprehensive representation of the complex aerodynamic interactions during dynamic stall.

The results depicted in Figure 6 serve as a testament to the reliability and versatility of the method employed, particularly the utilization of the user-defined function (UDF) across varying Reynolds numbers. Through this comprehensive analysis, encompassing different Reynolds numbers and corresponding aerodynamic phenomena, the efficacy of the proposed approach is underscored.

## 4. Conclusions

By providing a comprehensive exposition of the exact methodology for reproducing dynamic stall on any airfoil using a UDF and ANSYS Fluent, the primary objective of this paper is to facilitate a more accessible approach for venturing into the fields of aerodynamics and dynamic stall research.

In analyzing the results obtained using the presented method and juxtaposing them with findings from the literature, it becomes evident that variations in the turbulence model, airfoil size, range of AoA, and mesh quality contribute significantly to discrepancies between results. Nonetheless, upon closer examination, it is apparent that despite these discrepancies, all CFD models compared cover similar areas of results. This observation underscores the robustness and effectiveness of the method outlined in this paper.

The incorporation of the UDF presented within ANSYS Fluent not only allows for a higher degree of customization but also enhances the fidelity of results. By leveraging UDFs, the simulation methodology gains the capability to finely tune parameters, such as frequency, angle of attack range, and airfoil dynamics, resulting in a more tailored approach to investigating dynamic stall effects on airfoils. This level of customization enables simulations to capture intricate flow behaviors with greater detail, thereby improving the accuracy and reliability of the results. This approach allows for the seamless coupling of sophisticated aerodynamic analyses with customizable airfoil kinematics, enabling the investigation of complex flow interactions with confidence. As a result, while the flow calculations leverage the established capabilities of ANSYS Fluent, the controlled movement of the airfoil through the UDF ensures the fidelity and reliability of the simulation outcomes, providing valuable insights into aerodynamic performance. In essence, the utilization of UDFs in ANSYS Fluent serves to bolster the robustness and comprehensiveness of the simulation methodology.

This paper inherently aligns with the principles of biomimetics, as it leverages insights from nature’s efficient flyers, such as birds and insects, to optimize the design and performance of micro airfoils. By emulating the aerodynamic characteristics observed in biological systems, this paper aims to set the basis for developing airfoil designs that mimic natural wing shapes, surface textures, and flight dynamics. Through the investigation of dynamic stall phenomena, which are prevalent in avian flight, this research endeavors to apply biomimetic principles to enhance the maneuverability, stability, and efficiency of MAVs and drones. The integration of biomimetic insights into airfoil design and analysis represents a significant contribution to the field of bio-inspired engineering, where solutions inspired by nature are utilized to address complex engineering challenges. Overall, the exploration of dynamic stall in the context of biomimetics and aerodynamics presents promising opportunities for interdisciplinary research and technological advancement with broad societal implications.

## Figures and Tables

**Table 1:** Main UDF script for reproducing dynamic stall.

Main UDF Body	Additional Comments
***DEFINE_CG_MOTION(airfoil, dt, vel, omega, time, dtime)***	CG_MOTION—predefined ANSYS function by six arguments: - airfoil: the name of the UDF; - dt (dynamic thread), vel (linear velocity), omega (angular velocity), time (current time), dtime (time step) are variables passed by the ANSYS Fluent solver to the UDF Function.
***{***	
***float in_omega, t, in_T, Stop, alpha_min, alpha_max, var_omega;***	Declaring multiple variables in one statement: - in_omega: initial value for ω; - t, time; - in_T, initial period; - Stop, maximum time allowed for one complete iteration; - alpha_min, minimum AoA for the dynamic stall; - alpha_max, the maximum AoA for the dynamic stall; - var_omega, variable form of ω.
***var_omega = 0.0;***	Initialization of the variable ω.
***in_omega = 0.55;***	Initialization of the initial parameter ω.
***t=time;***	Time allocated by the ANSYS Fluent solver to the UDF under the variable t.
in_T = 2 * pi/in_omega;	The variable in_T defines how many seconds a cycle lasts.
***Stop = (in_T + Start);***	The parameter Stop defines the maximum time allowed for one complete iteration.
alpha_min = (0) * (pi/180);	The dynamic stall starts at 0° AoA.
alpha_max = (18) * (pi/180);	The dynamic stall stops at 18° AoA.
***if (t <= Start)***	Exit loop 1, without variable modification.
***{***	
***var_omega = 0;***	
***omega [0] = var_omega;***	
***}***	
***else{***	
***if (t > Start && t <= Stop)***	Exit loop 2, with variable modification.
***{***	
var_omega= ((0.5 * in_omega * (alpha_max- alpha_min)) * sin(in_omega * (t-Start)));	
***omega [0] = var_omega;***	
***}***	
***else{***	
***if (t > Stop)***	Exit loop 3, without variable modification.
***{***	
***var_omega = 0;***	
***omega [0] = var_omega;***	
***}***	
***}***	
***}***	
***}***	

**Table 2:** Comparison of the obtained numerical results against experimental results.

Method	Reynolds Number	AoA Range [min°–max°]	Observations
Present work	$5.2\times 10^{5}$	0°–18°	NACA 0012, c=150 mm
	$1.04\times 10^{6}$	0°–18°	NACA 0012, c=150 mm
Experimental—Reddy et al. [7]	$2.5\times 10^{6}$	5°–25°	NACA 0012, c=127 mm
Experimental—Surekha et al. [11]	$10^{5}$	−5°–25°	NACA 0012, c=160 mm
Experimental—McAlister et al. [5]	$2.5\times 10^{6}$	0°–20°	NACA 0012, c=76.2 mm

**Table 3:** Comparison of obtained results against other CFD results.

Method	Re	Turbulence Model	AoA Range [min°–max°]	Observations
Present work	$5.2\times 10^{5}$	DES	0°–18°	NACA 0012, c=150 mm
	$1.04\times 10^{6}$	DES	0°–18°	NACA 0012, c=150 mm
CFD—Fan et al. [24]	$3.2\times 10^{4}$	SST gamma	0°–18°	3D NACA 0012, c=35 mm
CFD—Baldan et al. [25]	$1.35\times 10^{5}$	URANS	−5°–25°	NACA 0012, c=150 mm

## Data Availability

Not applicable.
